# A Dual‐Polarization Programmable Metasurface for Green and Secure Wireless Communication

**DOI:** 10.1002/advs.202403624

**Published:** 2024-06-20

**Authors:** Zheng Xing Wang, Jun Wei Wu, Hui Xu, Jun Yan Dai, Shuo Liu, Qiang Cheng, Tie Jun Cui

**Affiliations:** ^1^ Institute of Electromagnetic Space Southeast University Nanjing 210096 China; ^2^ State Key Laboratory of Millimeter Waves School of Information Science and Engineering Southeast University Nanjing 210096 China; ^3^ Peng Cheng Laboratory Shenzhen Guangdong 518055 China; ^4^ Pazhou Laboratory (Huangpu) Guangzhou Guangdong 510555 China

**Keywords:** dual‐polarization, green, low cost, programmable metasurface, secure communication

## Abstract

Dual‐polarization programmable metasurfaces can flexibly manipulate electromagnetic (EM) waves while providing approximately twice the information capacity. Therefore, they hold significant applications in next‐generation communication systems. However, there are three challenges associated with the existing dual‐polarization programmable metasurfaces. This article aims to propose a novel design to address them. First, the design overcomes the challenge of element‐ and polarization‐independent controls, enabling more powerful manipulations of EM waves. Second, by using more energy‐efficient tunable components and reducing their number, the design can be nearly passive (maximum power consumption of 27.7 mW), leading to a significant decrease in the cost and power consumption of the system (at least two orders of magnitude lower than the power consumption of conventional programmable metasurfaces). Third, the design can operate in a broad bandwidth, which is attractive for practical engineering applications. Both the element and array of the metasurface are meticulously designed, and their performance has been carefully studied. The experiments demonstrate that 2D wide‐angle beam scanning can be realized. Moreover, secure communication based on directional information modulation can be implemented by exploiting the metasurface and an efficient discrete optimization algorithm, showing its programmable, multiplexing, broadband, green, and secure features.

## Graphical abstract

A novel design is introduced to address the three challenges in the dual‐polarization programmable metasurface. The proposed design allows for independent control of each element and polarization while also featuring low cost, low power consumption, and broad bandwidth. The application of the design in wireless communication is presented, demonstrating its distinctive features, including programmability, multiplexing, greenness, and enhanced security.

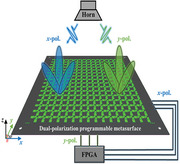

## 1 Introduction

From 1G to 5G, each generation of mobile communication systems introduces new technologies to improve service quality. Two critical technologies in 5G are millimeter wave and massive multiple‐input multiple‐output, and they have significantly boosted communication rates and system capacity, resulting in a superior network experience for users.^[^ ^1^, ^2^ ^]^ However, the increasing demand for enhanced service experience and vertical industry applications propels mobile communication systems forward. Academia and industry are initiating research on the 6G communication system.^[^ ^3^, ^4^ ^]^

As one of the pivotal technologies for 6G, programmable metasurfaces, also known as reconfigurable intelligent surfaces (RISs), have received widespread attention from researchers worldwide due to their flexibility in manipulating electromagnetic (EM) channels and environments.^[^ ^5^, ^6^, ^7^, ^8^, ^9^, ^10^, ^11^ ^]^ The programmable metasurfaces are 2D artificial materials composed of intricately designed elements.^[^ ^5^ ^]^ By applying control signals to the tunable components (such as positive intrinsic negative (PIN) diodes) on the elements, we can dynamically manipulate the magnitude,^[^ ^12^, ^13^, ^14^ ^]^ phase,^[^ ^15^, ^16^, ^17^ ^]^ polarization,^[^ ^18^, ^19^ ^]^ and frequency responses,^[^ ^20^ ^]^ thus programmatically reshaping EM wavefronts. In addition, the programmable metasurfaces have been gradually developed from the microwave frequency band to the terahertz band.^[^ ^21^, ^22^, ^23^ ^]^ The programmable metasurfaces have transformed wireless propagation from passive adaptation to active control, enabling the realization of smart radio environments.^[^ ^24^ ^]^ In addition, they feature low cost, low complexity, and easy deployment, providing significant benefits in improving wireless coverage and system capacity.^[^ ^25^, ^26^, ^27^ ^]^

Multiplexing techniques can be used with programmable metasurfaces to improve the multitasking and information‐processing capability of communication systems. Specifically, each polarization can be treated as an independent transmission channel. Hence, researchers have developed dual‐polarization programmable metasurfaces to achieve almost twice the information capacities. However, the existing dual‐polarization programmable metasurfaces face three fundamental challenges in practical engineering, and this article will propose a novel design approach to overcome them.

### 1.1 Challenge I: Independent Controls of Each Element and Polarization

The first challenge in designing dual‐polarization programmable metasurfaces is to achieve independent controls of each element and its polarization. The independent control of the elements results in substantial bias networks. Besides, their complexity is further increased due to the addition of the polarization channel. As a result, most of the existing dual‐polarization programmable metasurfaces are limited to just element design and have not been extended to the array level.^[^ ^28^, ^29^, ^30^, ^31^ ^]^

On the other hand, some dual‐polarization programmable metasurfaces are controlled in column (or row) to reduce the complexity of the bias networks.^[^ ^32^, ^33^ ^]^ Nevertheless, this comes at the cost of sacrificing the freedom to manipulate EM waves. For example, the column‐controlled 1‐bit programmable metasurface only offers dual‐beam scanning under plane wave excitation. The correlated beams will introduce unwanted noise and reduce the information capacity due to lower array gain. Moreover, their scanning is limited to only one dimension. Such limitations significantly restrict the application of metasurfaces in wireless communication systems. Therefore, designing metasurfaces with independent programmability in both element and polarization space is critical.

### 1.2 Challenge II: Low Cost and Power Consumption

Keeping cost and power consumption low is the other challenge in implementing dual‐polarization programmable metasurfaces. In practical applications, the programmable metasurfaces are usually very large, which leads to a significant increase in system overhead. Furthermore, pursuing “green” and “low carbon” has been a consistent goal of wireless communication systems. Although the power consumption of programmable metasurfaces is reduced compared to other technologies, such as traditional phased array antennas, they are still huge and become unacceptable (up to tens or even hundreds of watts) as the array size and polarization mode increase.

The cost and power consumption of the programmable metasurfaces are primarily due to the embedded PIN diodes. Currently, each element in dual‐polarization programmable metasurfaces requires at least four PIN diodes and consumes mW of power.^[^ ^28^, ^29^, ^34^ ^]^ Therefore, reducing power consumption and the number of tunable components while maintaining array performance will be a challenge in developing dual‐polarization programmable metasurfaces.

### 1.3 Challenge III: Broad Operating Bandwidth

The broad operating bandwidth enables high transmission rates and large data capacity for wireless communication, which is necessary for the practical application of dual‐polarization programmable metasurfaces. However, most existing dual‐polarization programmable metasurfaces can only work in a narrow bandwidth.^[^ ^34^, ^35^ ^]^ As the operating frequency shifts, their magnitude and phase responses will deteriorate. Therefore, we will overcome this challenge and propose a broadband design.

This article will introduce a novel design to address the three challenges in the dual‐polarization programmable metasurface. The proposed design allows for independent controls of each element and polarization while featuring low cost, low power consumption, and broad bandwidth. First, the metasurface element and array are designed. Then, 2D wide‐angle beam scanning in the two polarization channels is demonstrated. Finally, the application of the design in directional information‐modulated communication is presented, showing its distinctive features, such as programmability, multiplexing, broad bandwidth, greenness, and enhanced security.

## 2 Results

### 2.1 Element and Array Designs

**Figure 1:**
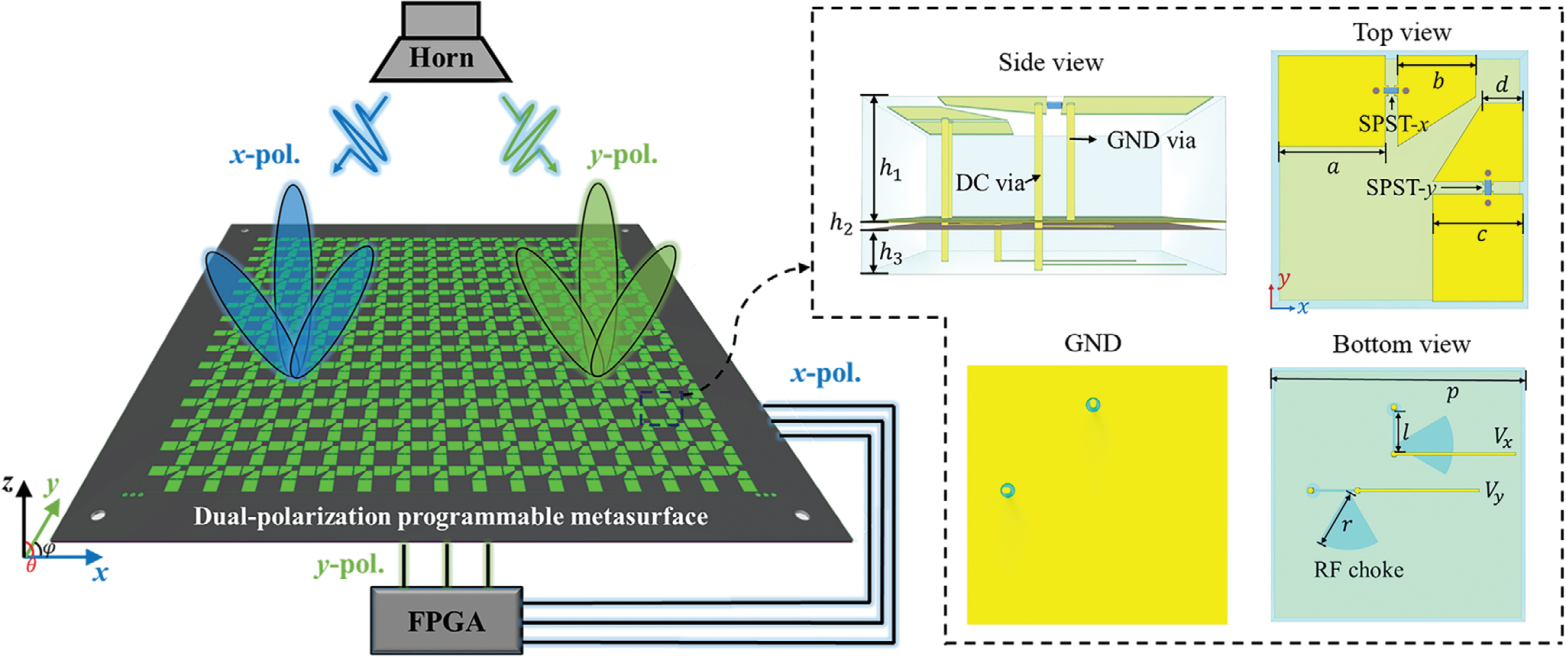
Proposed dual‐polarization programmable metasurface and its element structure. The key parameters are: *p* = 12.5 mm, *l* = 2.3 mm, *r* = 3 mm, *a* = 5 mm, *b* = 3 mm, *c* = 4 mm, *d* = 2 mm, *h*_1_ = 2.8 mm, *h*_2_ = 0.2 mm, *h*_3_ = 1 mm.

As shown in **Figure** **1**, the proposed dual‐polarization programmable metasurface consists of 16 ×  16 elements, each of which has a dimension of 12.5  ×  12.5 mm^2^, corresponding to an electrical size of 0.41  ×  0.41λ^2^ at 10 GHz. The element is composed of three parts: the top reflective layer, the middle metal ground layer, and the bottom direct current (DC) bias layer. The top layer comprises two identical patches along the *x*‐ and *y*‐directions etched on the surface of F4BTM265 substrate (ε_0_ =  2.65, tan δ =  0.0015). They are used to independently manipulate EM responses of the two linearly polarized incident waves. Each patch consists of rectangular and trapezoidal structures bridged by a single‐pole‐single‐throw (SPST) switch (ARW3172). Besides, bias networks are incorporated into each element to provide independent DC signal to the SPST switch. Taking the patch in the *x*‐direction as an example, there are two metal vias. The left and right ones are connected to the ground and the bias line on the bottom layer via an radio frequency choke (a microstrip sector patch), respectively. The patch along the *x*‐direction exhibits distinct equivalent resonant dimensions when the SPST‐*x* switch is either on or off, resulting in two different resonant frequencies. Therefore, two phase responses (0° and 180°) can be realized under the illumination of the *x*‐polarized wave. We use the digits “0” and “1” to encode the opposite phase responses of the patch. Then, by simply designing array coding sequences in this polarization channel, the programmable metasurface can achieve versatile functions.

To investigate the element, we perform full‐wave simulations in CST Microwave Studio. The boundary conditions of the element in both *x*‐ and *y*‐directions were unit cell, and the floquet port was utilized in the + *z*‐direction. Two excitation modes were used, corresponding to *x*‐ and *y*‐polarized wave incidence. Furthermore, the measured S‐parameter files (S2P files) of the SPST switch were imported into the software to perform the field‐circuit co‐simulation with the passive structure (the measurement of the S‐parameter of the SPST switch is presented in Note S1, Supporting Information). The element has four digital states, which are encoded as “0/0”, “0/1”, “1/0”, and “1/1”, respectively. The number before and after the slash corresponds to the phase response of the SPST‐*x* and SPST‐*y*, respectively. The numbers “0” and “1” represent the case when the SPST switch is turned on and off, respectively, and there is a theoretical 180° phase difference between them. Besides, the relationship between the four digital states and the bias voltage is summarized in **Table** **1**.

**Table 1:** The relationship between the digital states and the bias voltage.

State	0/0	0/1	1/0	1/1
Voltage				
*V_x_*	0 V	0 V	2.5 V	2.5 V
*V_y_*	0 V	2.5 V	0 V	2.5 V

**Figure 2:**
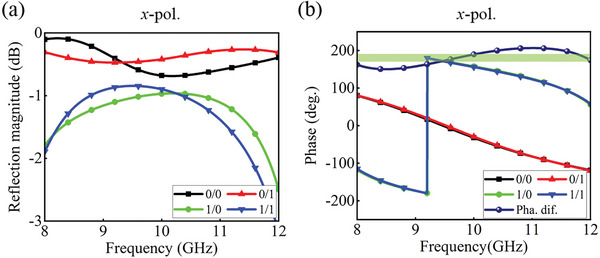
Simulation performance of the element. a) Reflection magnitude responses under the *x*‐polarized wave incidence. b) Phase responses under the *x*‐polarized wave incidence.

**Figure** **2** presents the magnitude and phase responses of the element under the *x*‐polarized wave incidence. The magnitude response is almost flat and greater than −1 dB in the 8–12 GHz when the element is under the “0/0” and “0/1” states. Besides, their phase curves agree, indicating good isolation between the two polarizations, and the change in one channel does not affect the other (as further demonstrated in Figure S2, Supporting Information). The magnitude response for the “1/0” and “1/1” states also remains almost the same, and their values are better than −1.5 dB in the 8.3–11.1 GHz range. The lower reflectivity in the two states can be attributed to the loss of the SPST switch. Similarly, their phase response curves overlap. In addition, the phase difference of the element remains within 180°  ±  30° throughout the frequency range, meeting the design requirements of 1‐bit programmable metasurfaces. To show the working mechanism of the element more intuitively, its surface current distribution at 10 GHz is plotted in Figure S3 (Supporting Information). Apparently, there are two different current distributions on the patch along the *x*‐direction for the “0/0” and “1/1” states, further demonstrating the existence of opposite phase responses. The situation is the same for the *y*‐polarized wave incidence. Since the incident wave is not always in the normal direction, it is necessary to evaluate the angular stability of the element. The magnitude and phase response of the element for the “1/0” state under the two linearly polarized waves with incident angles of 0°, 15°, and 30° were investigated (as presented in Note S4, Supporting Information). It is found that even under 30° wave incidence, performance variations of the element are still small. Therefore, we can conclude that the element shows strong angular robustness.

A dual‐polarization programmable metasurface is designed based on the elements. The metasurface is excited by a diagonal horn antenna operating in the X‐band. It is located 160 mm directly above the metasurface, corresponding to a focal‐to‐diameter ratio of 0.8. The beam steering performance of the programmable metasurface is first examined by full‐wave simulations. Since the 1‐bit phase quantization error is significant, the coding sequences of the metasurface are first optimized for each scanning angle. Simulation results demonstrate that the programmable metasurface can achieve 2D wide‐angle beam scanning in the two polarization channels (as shown in Figure S5, Supporting Information). For instance, considering the *x*‐polarization, the main beam of the metasurface can scan from −50° to 50°, with a gain loss of 2.4 and 1.8 dB in the E‐ and H‐planes, respectively. The gain of the broadside beam is 20.4 dBi, corresponding to 19.6% aperture efficiency. Additionally, the sidelobe level (SLL) for the E‐ and H‐planes is −13.4 and −16.6 dB, respectively. It is noteworthy that due to the blockage effect of the feed source, the maximum gain of the programmable metasurface does not occur at 0° but peaks at −20° with the value of 20.7 dBi, corresponding to 21% aperture efficiency.

### 2.2 Prototype Fabrication and Far‐Field Measurements

**Figure 3:**
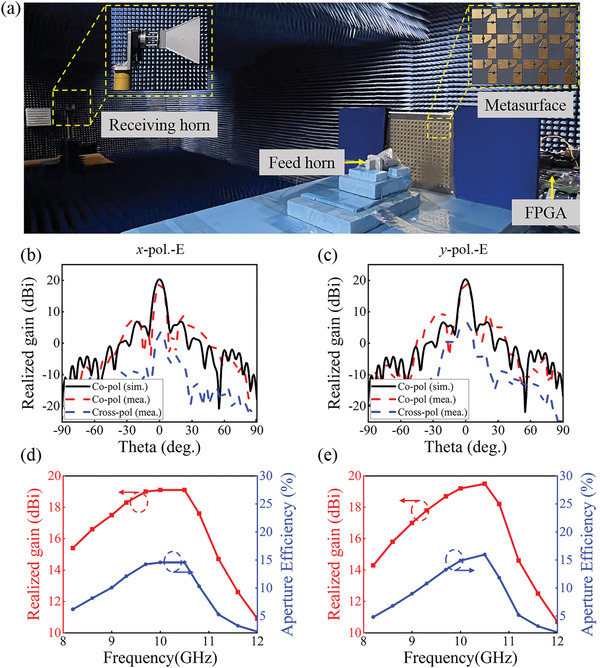
a) Far‐field measurements of the dual‐polarization programmable metasurface. b) Performance of the broadside beam at 10 GHz in the E‐plane for *x*‐polarization. c) Performance of the broadside beam in the E‐plane for *y*‐polarization. d) Gain and aperture efficiency bandwidth for *x*‐polarization. e) Gain and aperture efficiency bandwidth for *y*‐polarization.

To demonstrate the feasibility of the dual‐polarization programmable metasurface, a prototype was fabricated using standard printed circuit board technology (as presented in Figure S6, Supporting Information). The effective size of the programmable metasurface is 200 × 200 mm^2^ (electrical size of 6.67 × 6.67λ^2^ at 10 GHz), and the total size is increased to 240 × 240 mm^2^ for DC bias and assembly. The far‐field experiments are conducted in a microwave anechoic chamber, and the configuration is illustrated in **Figure** **3**a. The metasurface was connected to a field programmable gate array (FPGA) circuit board, and it received commands from the laptop via the I/O port. The diagonal and double‐ridged horns connected to ports 1 and 2 of a Vector Network Analyzer and served as transmitting and receiving antennas, respectively. The entire experimental setup was placed on a rotary table controlled by an external computer. The detailed beam scanning measurements are described in Note S7 (Supporting Information).

The performance of the broadside beam in the two polarization channels at 10 GHz was first investigated, and the measurements agree with the simulations. As shown in Figure 3b, the measured gain for the *x*‐polarized wave is 19.1 dBi, which is 1.3 dB lower than the simulated value. The SLL and cross‐polarization levels are also better than −10.6 and −15.6 dB, respectively. The situation is similar for the *y*‐polarized wave, as exhibited in Figure 3c. The gain and aperture efficiency of the broadside beam at different frequencies are presented in Figure 3d,e to demonstrate the broadband characteristics of the programmable metasurface. It can be seen that the programmable metasurface has a relatively stable gain in the range of 8.8–11 GHz (a relative bandwidth of 22%), with a fluctuation better than 3 dB.

The beam scanning is realized by changing the phase distributions (or coding sequences) on the programmable metasurface. The required compensation phase can be calculated as follows:

(1) $$\varphi_{rmn}=\varphi_{fmn}\;-k\times {\hat{u}}_{0}\times {\vec{r}}_{mn}+\Delta \varphi$$

where φ_*fmn*_ represents the phase shift caused by the path difference from the feed source to the (*m*, *n*)th element, *k* is the wave number in free space, ${\hat{u}}_{0}$ is the unit direction vector of the reflected beam, and ${\vec{r}}_{mn}$ is the position vector from the (*m*, *n*)th element to the center of the array. Δφ is an additional design freedom to optimize the phase distribution on the array. After calculating the continuous phase distribution φ_*rmn*_ in the intended direction, we discretize it according to the following equation to obtain the 1‐bit phase as well as the coding sequence:

(2)

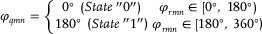




**Figure 4:**
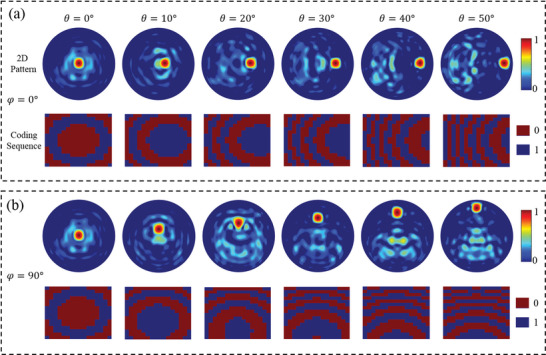
Far‐field patterns and their corresponding coding sequences at 10 GHz for *x*‐polarization. a) The main beam scans from 0° to 50° in the E‐plane. b) The main beam scans from 0° to 50° in the H‐plane.

**Figure 5:**
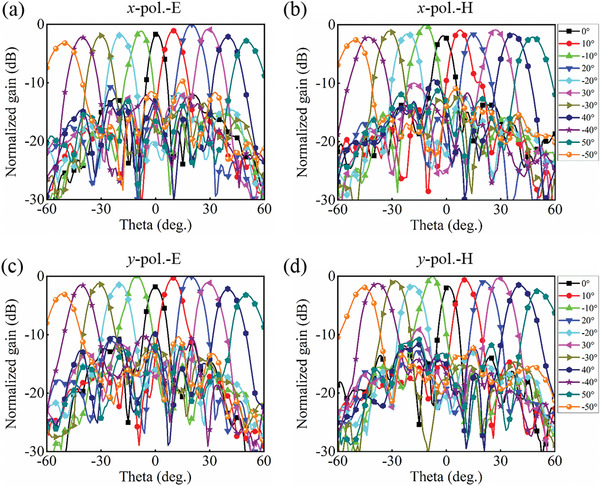
Measured beam scanning performance at 10 GHz. a) E‐plane for *x*‐polarization. b) H‐plane for *x*‐polarization. c) E‐plane for *y*‐polarization. d) H‐plane for *y*‐polarization.

Using *x*‐polarization as an example, the coding sequences on the metasurface and the corresponding far‐field patterns with different θ (positive angles) and φ are given in **Figure** **4**. The measured beam scanning performance of the programmable metasurface is presented in **Figure** **5**. The experiments show that the main beam can scan from −50° to 50° in both polarization channels with a step of 10°. For *x*‐polarized wave incidence, the scan gain loss in the E‐ and H‐planes is 3.2 and 2.6 dB, respectively. Similarly, the maximum gain pointing angle is not 0° due to the blockage effect of the feed source and coaxial line. To better compare the simulations and measurements, the gain and aperture efficiency of each scanning angle in the principal planes for the two polarization channels are summarized in Figure S8 (Supporting Information).

Since the proposed dual‐polarization programmable metasurface uses only two SPST switches for each element, and each switch consumes little energy, the power consumption is significantly reduced. The maximum value is only 27.7 mW (as shown in Figure S9, Supporting Information), at least two orders of magnitude lower than the power consumption of conventional programmable metasurfaces.^[^ ^27^, ^31^, ^34^ ^]^ The nearly passive power consumption greatly facilitates the large‐scale application of programmable metasurfaces in green wireless communication.

### 2.3 Application for Secure Wireless Communication

Eavesdropping protection of wireless communication systems has always been a formidable challenge—traditional communication schemes broadcast information across the whole space without discrimination. The fields in various directions vary only in signal‐to‐noise ratio but inherently carry the transmitted information, thus posing inherent security risks. While encrypting data at the network layer can improve information security, it comes at the cost of lengthening message code and increasing transmission overhead. In addition, the encryption and decryption process is complicated, making it challenging to meet the high speed and low latency requirements of communication systems.^[^ ^36^ ^]^

Directional information modulation is a physical layer security technology with a bright prospect. The innovative approach can generate accurate constellation symbols in desired directions while intentionally distorting them in other directions, effectively addressing issues related to malicious eavesdropping and information security.^[^ ^37^ ^]^ Several communication schemes based on directional information modulation have been proposed, primarily using linear phased or time‐modulated arrays.^[^ ^38^, ^39^ ^]^ However, they suffer from bulky size, high cost, and high power consumption. Moreover, they lack support for 2D space and polarization multiplexing.

To overcome these limitations, we applied the proposed dual‐polarization programmable metasurface in wireless communication based on directional information modulation and demonstrated its programmable, multiplexing, green, and secure features. We considered scenarios involving single and dual users as proof‐of‐concept illustrations and performed 8 phase shift keying (8PSK) and quadrature phase shift keying (QPSK) constellation diagram transmissions in *x*‐ and *y*‐polarization channels. Note that although the 1‐bit programmable metasurface initially has only two digital states, the independent controllability of each element allows its phase difference to the user to be optimized. Such a process introduces additional degrees of freedom and thus enables higher‐order information modulation.

To obtain the coding sequences on the metasurface, we consider the following situation: A dual‐polarization programmable metasurface in the *xoy* plane containing *N* digital elements serves *K* users simultaneously by transmitting constellation symbols to each of them. The reference constellation sets include 8PSK and QPSK (noted as O). Then, the *k*th user receives a signal as

(3) $$y_{k}=h_{k}^{H}\;x+n,\;k\;=\;1,\;\text{…},\;K$$

where $x\in C^{N}$ and $n∼CN(0,\;\sigma^{2})$ are the coding sequences and the complex additive white Gaussian noise, respectively. $h_{k}^{H}\in C^{N}$ is the channel vector and can be expressed as:

(4) $$h_{k}^{H}=e^{-jd_{k}}\;\sqrt{G\left(\theta_{k},\;\varphi_{k}\right)}{\left[e^{jv_{1,1}^{H}u},\;\text{…},\;e^{jv_{p,q}^{H}u},\;\text{…},\;e^{jv_{N_{x},N_{y}}^{H}u}\right]}^{T}$$

where *d_k_* and *G*(θ_*k*_, φ_*k*_) are the distance and gain of the element for the *k*th user, respectively. ***v***_*p*,*q*_ =  [*pd_x_*, *qd_y_*] and ***u***  =  2π/λ[*sin*θ_*k*_ *cos*φ_*k*_, *sin*θ_*k*_ *sin*φ_*k*_]^*T*^ are the auxiliary vectors used to simplify Equation 4. The goal of the optimization is to minimize the difference between the received signals ***y***  =  [*y*_1_, …,  *y_k_*]^*T*^ and the reference signals $s\;={\left[s_{1},\;\text{…},\;s_{k}\right]}^{T}\;\in O$. Therefore, the problem to be solved can be written as

(5) $$\min_{x,\;\tilde{x}}\;{\left\|s-Hx\right\|}^{2}+K\sigma^{2}$$

(6) $$s.\;t.\;\begin{array}{c} \;\tilde{x}=\;x\\ \tilde{x}\left(i\right)\in X,\;i\;=\;1,\;\text{…},\;N \end{array}$$

where $H\;=\;\left[h_{1}^{H},\;h_{2}^{H},\;\text{…},\;h_{K}^{H}\right]\in C^{K\times N}$, $\tilde{x}$, and $X\;=\{1/\sqrt{N}e^{jw_{i}}|w_{i}=2\pi i/2^{Q},\;i=1,2,\;\text{…},\;2^{Q}\}\;$ are the channel matrix, the auxiliary variable, and the *Q*‐bit phase quantization set, respectively. Here, a fast and efficient algorithm under the alternating direction method of multipliers (ADMM) was used to optimize the coding sequences in both polarization channels.^[^ ^40^, ^41^ ^]^ The algorithm has fast convergence and stability features and can decompose a non‐convex problem into several convex problems. The optimization process is described in Note S10 (Supporting Information). After obtaining the coding sequences, the FPGA converted them into the appropriate voltages to control the programmable metasurface.

**Figure 6:**
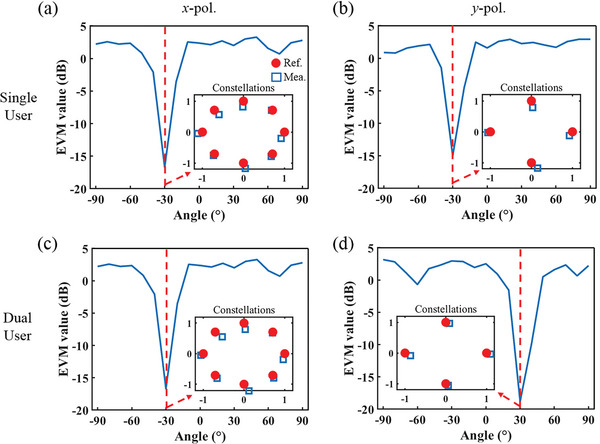
EVM values in single‐ and dual‐user mode, where the measured constellation symbols are in the intended direction. The red circular marker represents the reference symbols. a) *x*‐polarized channel in single‐user mode. b) *y*‐polarized channel in single‐user mode. c) *x*‐polarized channel in dual‐user mode. d) *y*‐polarized channel in dual‐user mode.

The programmable metasurface was first configured to operate in single‐user mode, transmitting 8PSK and QPSK symbols in the desired direction of (θ_1_, φ_1_)  =  (− 30°,  180°) under the incidence of *x*‐ and *y*‐polarized waves. The magnitude and phase of the signals in each direction were measured using an experimental setup similar to the beam scanning measurements. The measured values in the user direction are shown in Figure S10 (Supporting Information) and are in good agreement with the reference. **Figure** **6**a,b shows the corresponding constellation diagrams, and it can be seen that the measurements agree well with the reference symbols for both polarized waves. The results of other undesired directions are also plotted in Figure S11 (Supporting Information), and it can be obtained that the received signals are distorted. To further demonstrate the secure features of the communication scheme, the error vector magnitude (EVM) values for different angular directions were calculated (the definition of EVM can be found in Note S13, Supporting Information). We can observe that the EVM is sufficiently low only in the vicinity of the user direction. At the same time, it is high in other directions, indicating the directional nature of the communication scheme. To further demonstrate the security of the proposed communication scheme in the entire upper half space, the relationship of EVM values with θ and φ has been calculated, and the results in single‐user mode are presented in Figure S12 (Supporting Information).

**Figure 7:**
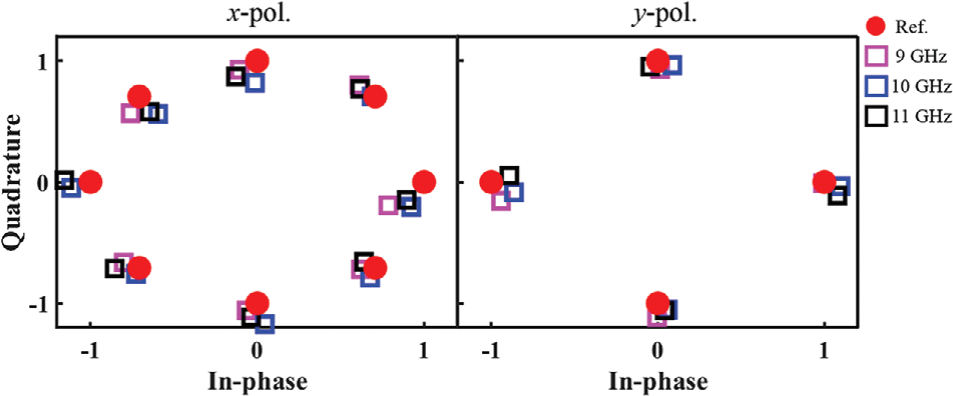
The broadband performance of the programmable metasurface in dual‐user mode. The red circular marker represents the reference symbols. The magenta, blue, and black rectangular markers represent the measured symbols at 9, 10, and 11 GHz, respectively.

To validate the 2D space multiplexing capabilities of the scheme, the programmable metasurface was configured to work in dual‐user mode, where it transmitted 8PSK symbol in the direction of (θ_1_, φ_1_)  =  (− 30°,  180°) using *x*‐polarized wave and QPSK symbol in the direction of (θ_2_, φ_2_)  =  (30°,  90°)using *y*‐polarized wave. The measured constellation diagrams in the intended directions are plotted in Figure 6c,d, which match well with the reference symbols (the corresponding magnitude and phase of the signals are shown in Figure S14, Supporting Information). However, the received signals are distorted in other directions (as shown in Figure S15, Supporting Information). Similarly, the EVM values in this mode were calculated (as shown in Figure 6c,d; Figure S13, Supporting Information). The results show that good constellation diagrams can only be received in the two intended directions. Finally, to demonstrate the broadband properties of the scheme, the symbols at different frequencies were measured. As shown in **Figure** **7**, the received signals show good agreement with the reference from 9 to 11 GHz.

To illustrate the advantages of the programmable metasurface, a list of key performance indicators is provided in **Table** **2**, which compares this work to other state‐of‐the‐art designs. It can be observed that the proposed programmable metasurface can realize independent controls of each element and polarization. Moreover, it has absolute advantages in terms of power consumption and bandwidth. Last but not least, the proposed metasurface can realize secure communication by exploiting an efficient discrete optimization algorithm.

**Table 2:** . Performance comparison of the proposed metasurface with state‐of‐the‐art designs.

Performance	Element control	Polarization control	Power consumption	Realtive bandwidth	Secure communication
Reference					
[27]	No	Single	26.6 mW/unit	Almost single frequency	Yes
[31]	No	Dual but not independent	26.6 mW/unit	10.5%	No
[32]	No	Dual and independent	–	Almost single frequency	No
[34]	Yes	Dual and independent	34 mW/unit	11.6%	No
This work	Yes	Dual and independent	0.1 mW/unit	22%	Yes

## 3 Conclusion

A novel design was introduced to address three challenges in the dual‐polarization programmable metasurface. First, its elements can be individually controlled with dual polarization reconfigurability, providing great flexibility in manipulating EM waves. Second, it significantly reduces the power consumption. The maximum value is only 27.7 mW, which is at least two orders of magnitude lower than that of conventional programmable metasurfaces, resulting in a low‐cost and nearly passive system. Third, it exhibits stable performance across a broad bandwidth to facilitate practical applications. The measurements indicate that the metasurface enables 2D wide‐angle beam scanning under the incidence of *x*‐ and *y*‐polarized waves. Furthermore, secure communication based on directional information modulation can be implemented by exploiting the metasurface and an efficient discrete optimization algorithm, showing its programmable, multiplexing, broadband, green, and secure features. The proposed design and scheme will play a significant role in the new green and secure communication system regime.

## Conflict of Interest

The authors declare no conflict of interest.

## Supporting information

Supporting Information

## Data Availability

The data that support the findings of this study are available from the corresponding author upon reasonable request.
