# Supplementary material for: A Dual‐Polarization Programmable Metasurface for Green and Secure Wireless Communication
Source: Adv Sci (Weinh). 2024 Jun 20;11(32):2403624. doi: 10.1002/advs.202403624 (PMC11348053; doi:10.1002/advs.202403624)
Supplement: Supplementary file 1 — Supporting Information [file ADVS-11-2403624-s001.docx]

**Supplementary Information for**

A Dual-Polarization Programmable Metasurface for Green and Secure Wireless Communication

Zheng Xing Wang^1, 2, †^, Jun Wei Wu^1, 2, 3, 4, †^, Hui Xu^1, 2^, Jun Yan Dai^1, 2^, Shuo Liu^1, 2^, Qiang Cheng^1, 2, 3, 4,^ *, Tie Jun Cui^1, 2, 3, 4,^ *

^1^ *Institute of Electromagnetic Space, Southeast University, Nanjing, 210096, China*

^2^ *State Key Laboratory of Millimeter Waves, School of Information Science and Engineering, Southeast University, Nanjing 210096, China*

^3^ *Peng Cheng Laboratory, Shenzhen, Guangdong 518055, China*

^4^ *Pazhou Laboratory (Huangpu), Guangzhou, Guangdong 510555, China*

†Equally contributed to this work.

*Email: qiangcheng@seu.edu.cn; tjcui@seu.edu.cn

**The supplementary information includes:**

- **Note 1. Measurement of the S-parameter of the SPST switch**
- **Note 2. Polarization isolation performance of the element**
- **Note 3. Surface current distribution of the element**
- **Note 4. Angular stability of the element**
- **Note 5. Simulated beam scanning performance of the programmable metasurface**
- **Note 6. The fabricated programmable metasurface prototype**
- **Note 7. The detailed beam scanning measurements**
- **Note 8. Comparison of simulated and measured beam scanning performance**
- **Note 9. Actual power consumption of the programmable metasurface**
- **Note 10. Details of the optimization process based on the ADMM algorithm**
- **Note 11. The measurement and reference signals for single-user mode**
- **Note 12. Constellation diagrams in other directions for single-user mode**
- **Note 13. Definition of EVM**
- **Note 14. Relationship of EVM values with** $\boldsymbol{\theta}$ **and** $\boldsymbol{\varphi}$
- **Note 15. The measurement and reference signals for dual-user mode**
- **Note 16. Constellation diagrams in other directions for dual-user mode**

**Note 1. Measurement of the S-parameter of the SPST switch**

Figure S1 shows the actual test scenario for the S-parameter of the SPST switch. The specific test steps are summarized below:

- Turn on the Vector Network Analyzer (VNA), connect the coaxial lines, and enter the calibration interface;
- Select the appropriate calibration method and calibrator type;
- Connect the calibrators (Open, Short, Load, Line-1, Line-2, Line-3, Thru) to complete the calibration;
- Place the SPST switch to be tested and press it carefully;
- Turn on the DC supply, adjust the voltage, and record the data;
- Complete the test, turn off the instruments, and export the data.

**
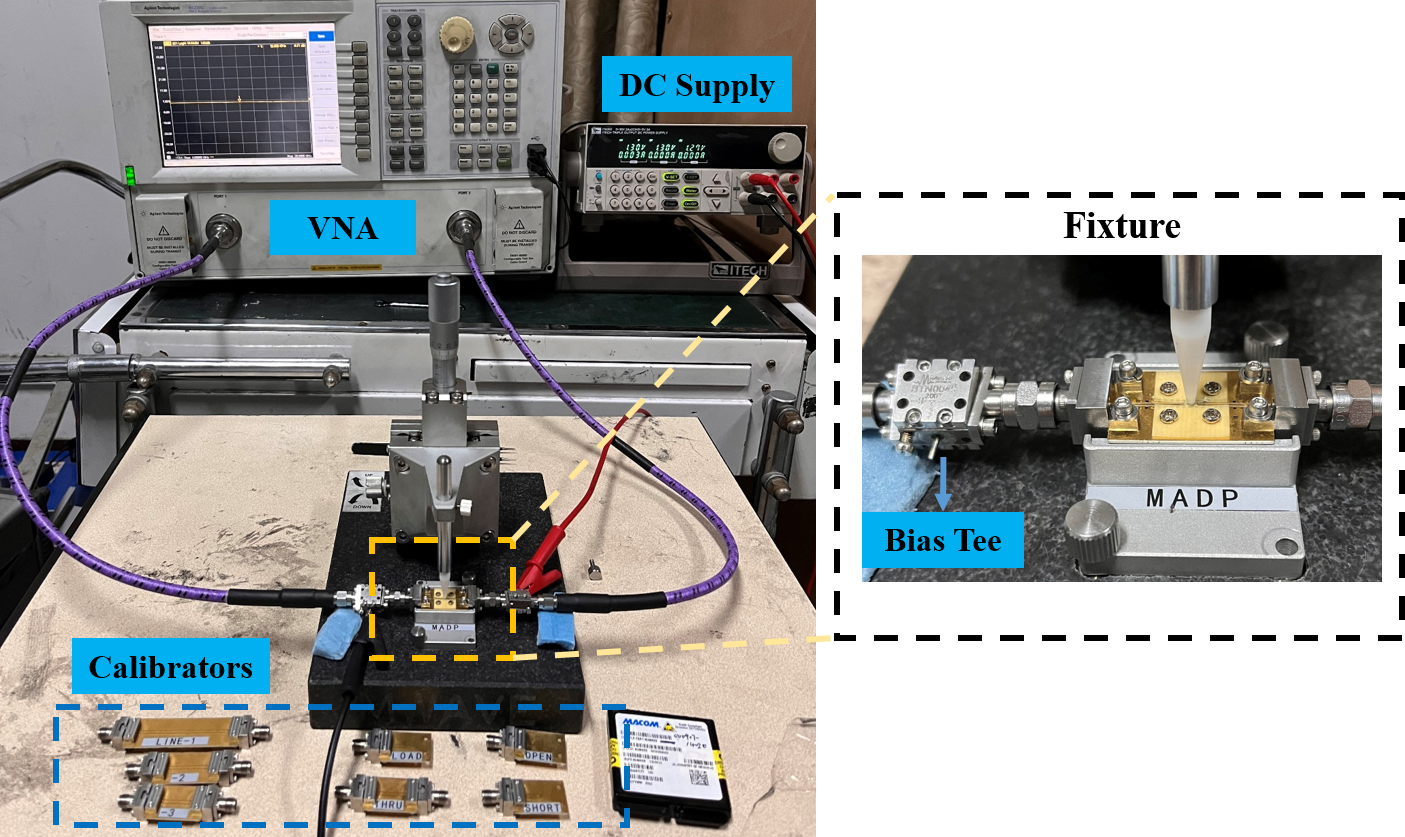
**

**Figure S1.** Actual test scenario for the S-parameter of the SPST switch.

**Note 2. Polarization isolation performance of the element**

The polarization isolation performance of the element under the four digital states was investigated. The results are shown in Figure S2, where $S_{yx}$ denotes *x*-polarized wave incidence and *y*-polarized wave reception. It can be seen that the isolation between the two polarization channels is less than -10 dB in the 8~12 GHz frequency band.

**
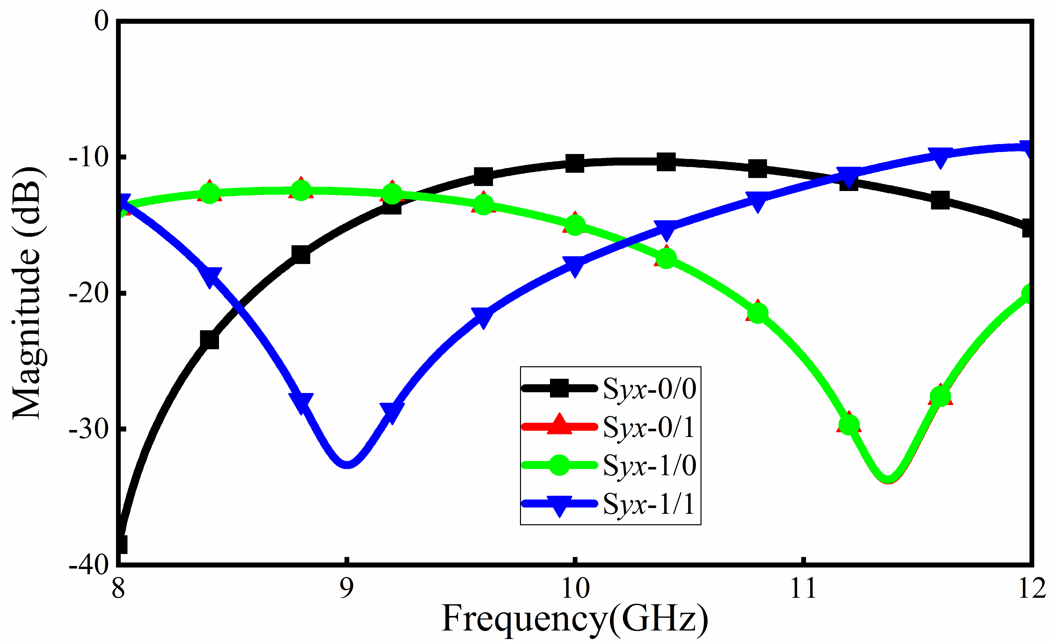
**

**Figure S2.** The polarization isolation performance of the element under the four digital states.

**Note 3. Surface current distribution of the element**

To show the working mechanism of the element more intuitively, its surface current distribution at 10 GHz is plotted in Figure S3. It can be obtained that there are two different current distributions on the patch along the *x*-direction, further demonstrating the existence of opposite phase responses of the element.

**
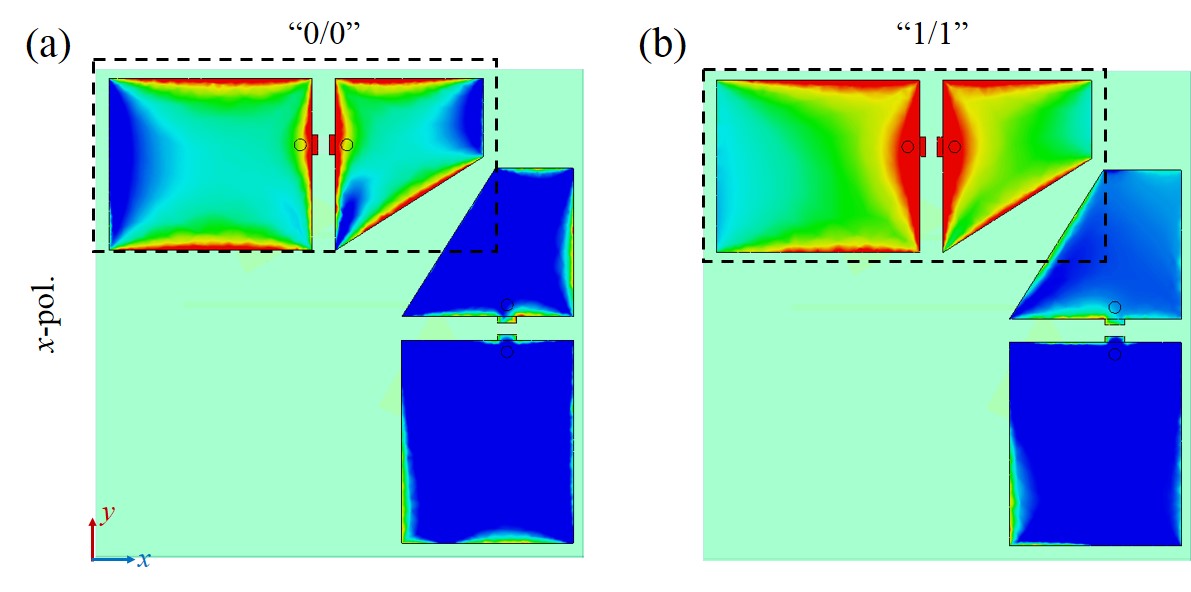
**

**Figure S3.** The surface currents of the element at 10 GHz under the illumination of *x*-polarized wave. (a) “0/0” state. (b) “1/1” state.

**Note 4. Angular stability of the element**

Since the excitation signal of the metasurface is not always at normal incidence, it is necessary to investigate the angular stability of the element. Using the element under the “1/0” digital state as an example, the magnitude and phase responses for the two linearly polarized waves with incident angles of 0°, 15°, and 30° were investigated. As shown in Figure S4, it is found that even under 30° wave incidence, the performance variations of the element are still small. Therefore, it can be concluded that the element exhibits strong angular robustness.

**
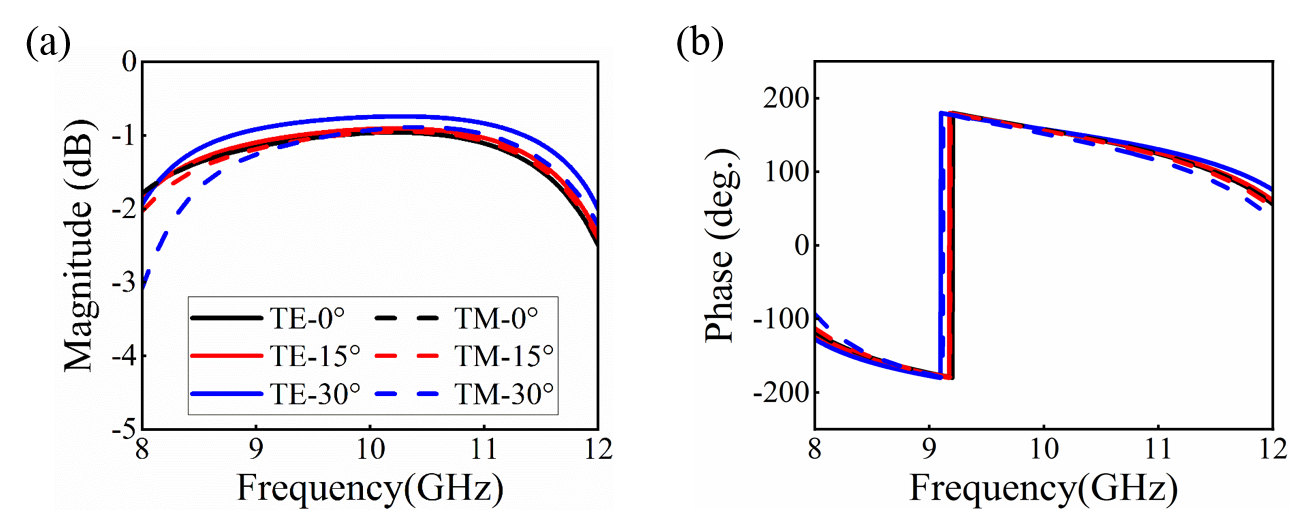
**

**Figure S4.** Angular stability of the element under the “1/0” states. (a) Amplitude response of the element at different angles. (b) Phase response of the element at different angles.

**Note 5. Simulated beam scanning performance of the programmable metasurface**

The simulated beam scanning performance of the programmable metasurface at 10 GHz is presented in Figure S5. For both polarization channels, the metasurface can scan from -50° to 50°. Considering the *x*-polarization, the gain of the broadside beam is 20.4 dBi, with a scan gain loss of 2.4 dB and 1.8 dB in the E- and H-planes, respectively. Besides, the sidelobe level (SLL) for the two principal planes is -13.4 dB and -16.6 dB, respectively.


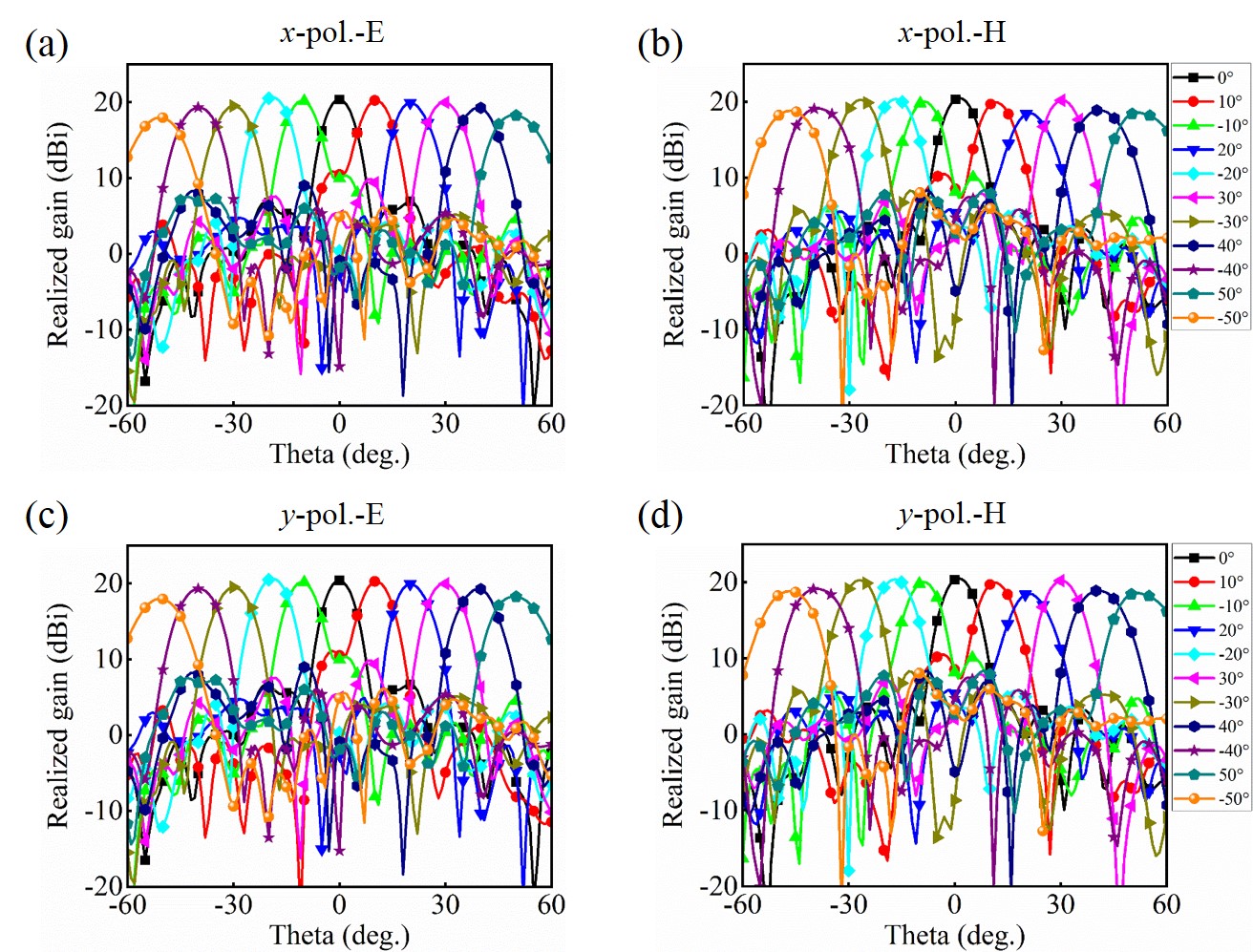


**Figure S5.** Simulated beam scanning performance of the programmable metasurface at 10 GHz. (a) E-plane for *x*-polarization. (b) H-plane for *x*-polarization. (c) E-plane for *y*-polarization. (d) H-plane for *y*-polarization.

**Note 6. The fabricated programmable metasurface prototype**

The dual-polarization programmable metasurface was fabricated and shown in Figure S6. Its effective size is 200 $\times$ 200 mm^2^ (electrical size of 6.67 $\times$ 6.67$\lambda^{2}$ at 10 GHz), and the total size is increased to 240 $\times$ 240 mm^2^ for DC bias and assembly. The metasurface consists of 256 elements, each containing two SPST switches.


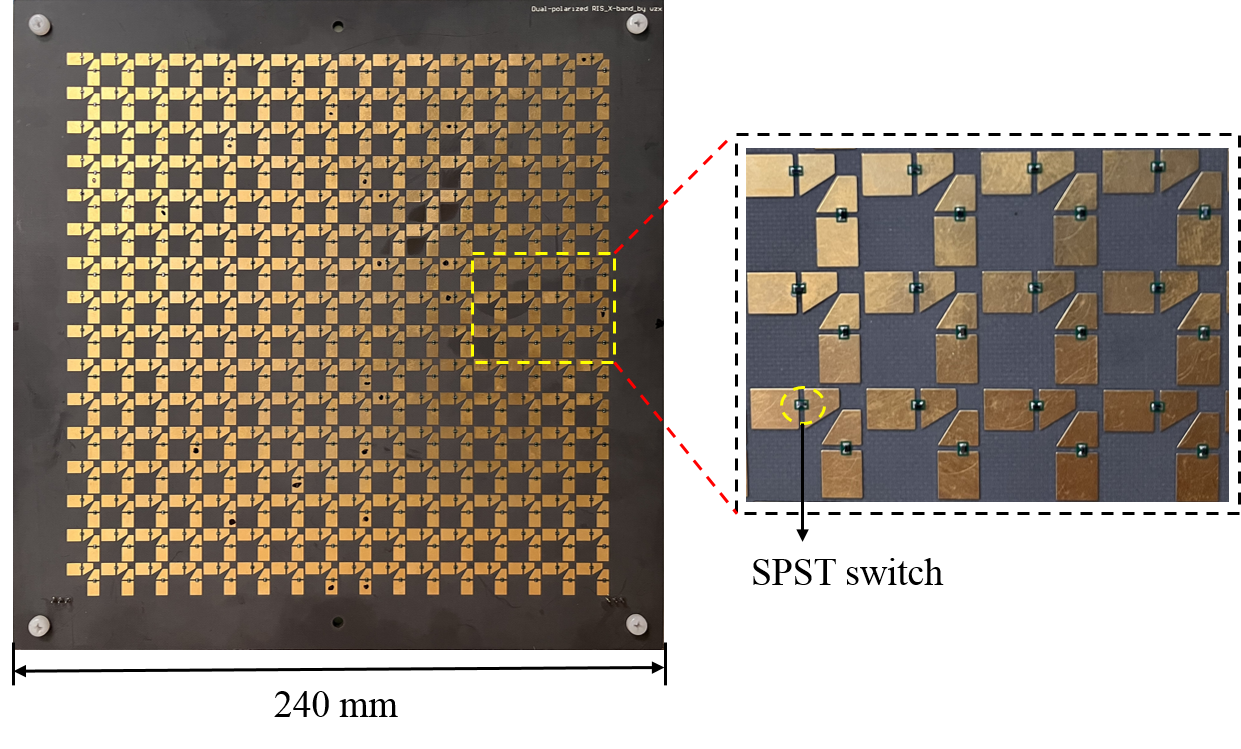


**Figure S6.** The fabricated prototype of the dual-polarization programmable metasurface.

**Note 7. The detailed beam scanning measurements**

As shown in Figure S7, the transmitting antenna is connected to one port of a vector network analyzer (VNA), which excites the metasurface. The antenna and the metasurface are placed on a rotary table that moves from -90° to 90° in the *xoz* plane at a speed of 1°/sec. The receiving antenna is connected to the other port of the VNA, which picks up the signals reflected from the metasurface. Then, the measurement results of different $\theta$ when $\varphi$ is 0° (E-plane) are obtained. Next, we simultaneously rotate the metasurface and the transmitting antenna by 90° around the *z*-axis and repeat the above tests, obtaining the results under $\theta$ along $\varphi$ $=$ 90° (H-plane).

**
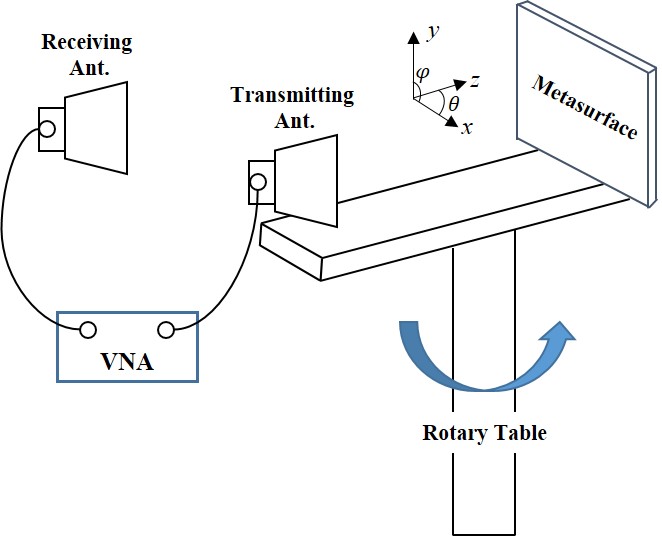
**

**Figure S7.** The far-field measurement setup.

**Note 8. Comparison of simulated and measured beam scanning performance**

To better compare the simulated and measured beam scanning performance, the gain and aperture efficiency of each scanning angle in the principal planes for the two polarization channels are plotted in Figure S8. It can be seen that the measurements are in good agreement with the simulations. For some scanning angles, the measurements are larger than the simulations due to the multipath effect (including reflection from the feed source and the coaxial line) in the experiments.

**
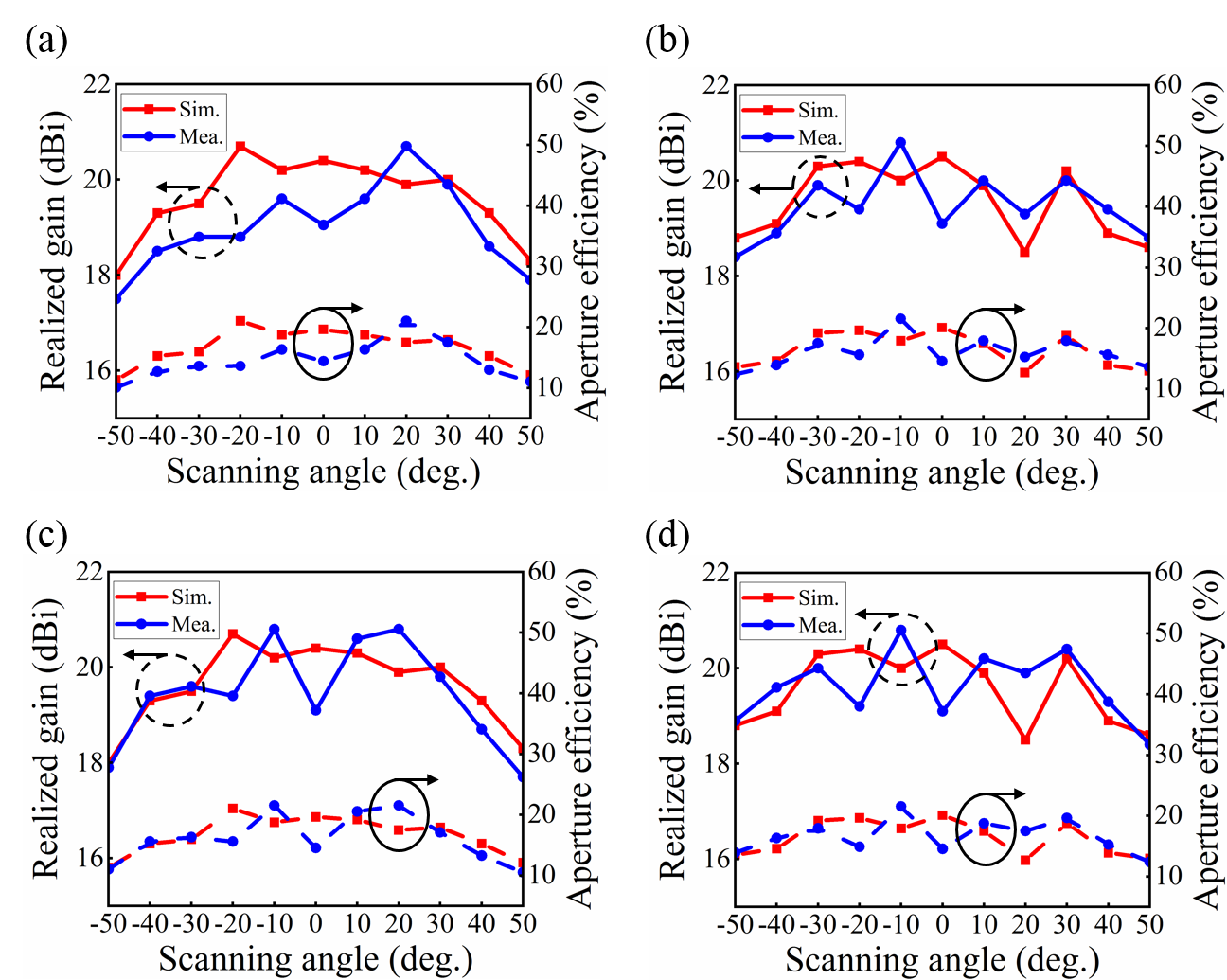
**

**Figure S8.** Simulated and measured gain and aperture efficiency at 10 GHz for each scanning angle. (a) E-plane for *x*-polarization. (b) H-plane for *x*-polarization. (c) E-plane for *y*-polarization. (d) H-plane for *y*-polarization.

**Note 9. Actual power consumption of the programmable metasurface**

The actual test scenario for the metasurface power consumption is presented in Figure S9. A digital multimeter (model FLUKE 12E+) was connected in series between the control circuit board (128 DC control signals) and the metasurface. The measurement shows that the consumed current is 2.77 mA. Therefore, the maximum power consumption of the metasurface (512 DC control signals) is 27.7 mW (2.77 mA*2.5 V*4=27.7 mW).

**
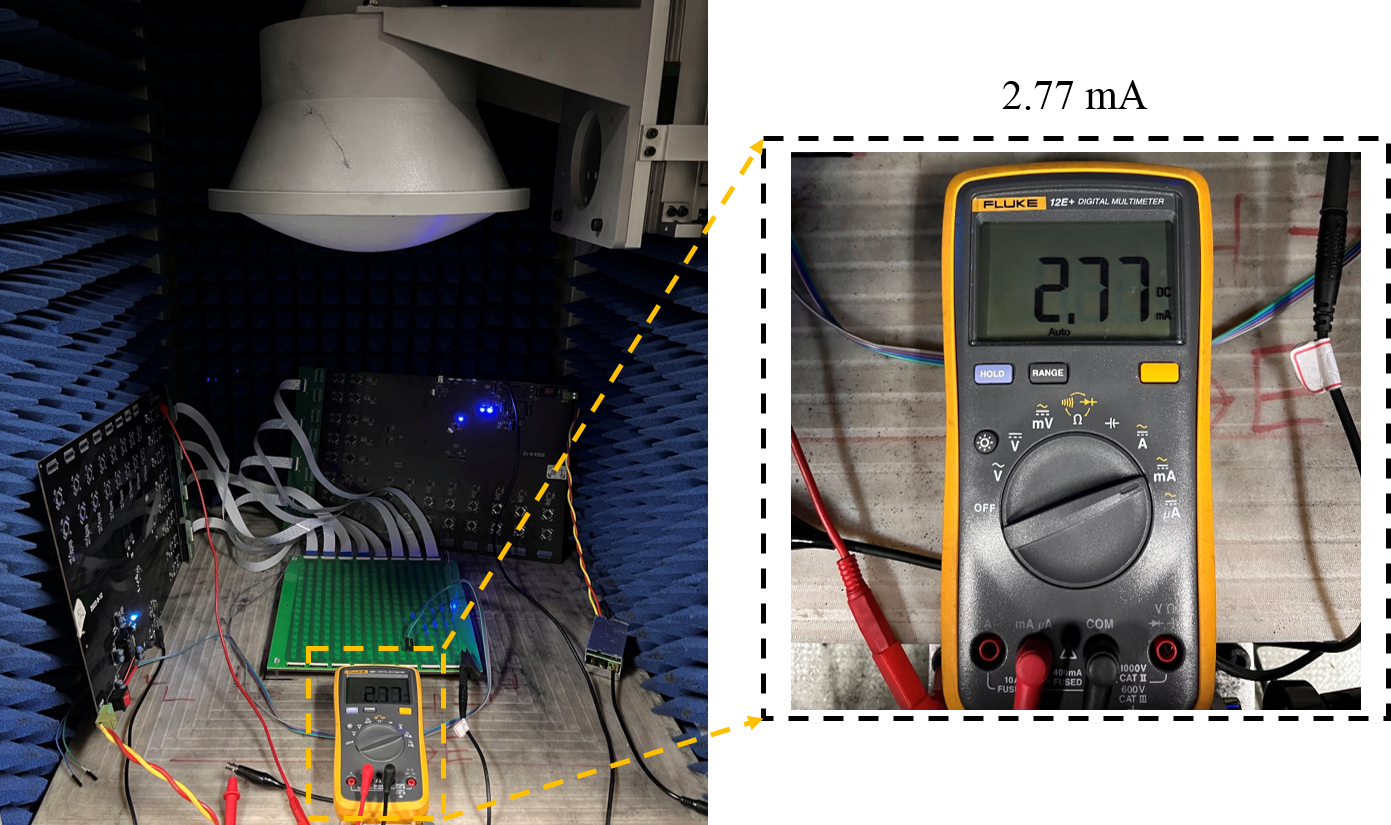
**

**Figure S9.** Actual test scenario for the metasurface power consumption.

**Note 10. Details of the optimization process based on the ADMM algorithm**

Here, we solved the problem using the ADMM algorithm. First, the augmented Lagrangian function for the problem of equation (3) can be written as

$\mathcal{L}(\boldsymbol{x}, \tilde{\boldsymbol{x}}, \text{u})=\left\| \boldsymbol{s}-\mathbf{H}\boldsymbol{x} \right\|^{2}+K\sigma^{2}+\mathcal{R}\left\{ \boldsymbol{u}^{H}(\tilde{\boldsymbol{x}}-\boldsymbol{x}) \right\}+0.5\rho\left\| \tilde{\boldsymbol{x}}-\boldsymbol{x} \right\|^{2},$ (S1)

where $\boldsymbol{u}\in\mathbb{C}^{N}$and $\rho$ are the dual vector and penalty parameters, respectively. The problem can be decomposed into several subproblems by using the alternating optimization strategy

${\tilde{\boldsymbol{x}}}^{t+1}=\underset{\tilde{\boldsymbol{x}}}{arg min} \mathcal{L}(\boldsymbol{x}^{t}, \tilde{\boldsymbol{x}}, \boldsymbol{u}^{t}),$ (S2)

$\boldsymbol{x}^{t+1}=\underset{\boldsymbol{x}}{arg min} \mathcal{L}(\boldsymbol{x}, {\tilde{\boldsymbol{x}}}^{t+1}, \boldsymbol{u}^{t}),$ (S3)

$\boldsymbol{u}^{t+1}=\boldsymbol{u}^{t}+\rho({\tilde{\boldsymbol{x}}}^{t+1}-\boldsymbol{x}^{t+1}).$ (S4)

Both equations (S2) and (S3) have closed-form solutions and can be expressed as follows

${\tilde{\boldsymbol{x}}}^{t+1}=1/\sqrt{N}[e^{j\tilde{w}_{1}^{t+1}}, e^{j\tilde{w}_{2}^{t+1}}, ..., e^{j\tilde{w}_{N}^{t+1}}]^{T},$ (S5)

$\boldsymbol{x}^{t+1}=0.5(\mathbf{R}_{H}^{t})^{-1}\phi^{t+1},$ (S6)

where $\tilde{w}_{i}^{t+1}$ is the optimized discrete phase of the $i$th digital element in the $t+1$ iteration, $\mathbf{R}_{H}^{t}=\mathbf{H}^{H}\mathbf{H}+0.5\rho\mathbf{I}_{N}$, and $\phi^{t+1}=2\mathbf{H}^{H}\boldsymbol{s}+\boldsymbol{u}^{t}+\rho{\tilde{\boldsymbol{x}}}^{t+1}$. Thus, the optimization proceeds as follows

- **Input**: $\boldsymbol{x}^{(0)}$, $\boldsymbol{u}^{(0)}$, $\sigma^{2}$, $\rho$, $T$
- **For** $t=0$ **to** $T$

Update ${\tilde{\boldsymbol{x}}}^{t+1}$, $\boldsymbol{x}^{t+1}$, and $\boldsymbol{u}^{t}$

$t=t+1$

- **End**
- **Output:** $\boldsymbol{x}=\boldsymbol{x}^{T}$

**Note 11. The measurement and reference signals for single-user mode**

In single-user mode, the amplitude and phase of the measurement and reference signals in the user direction of $(\theta_{1}, \varphi_{1})=(-30^{\circ}, 180^{\circ})$are plotted in Figure S10. It can be seen that the measurements are in good agreement with the reference.


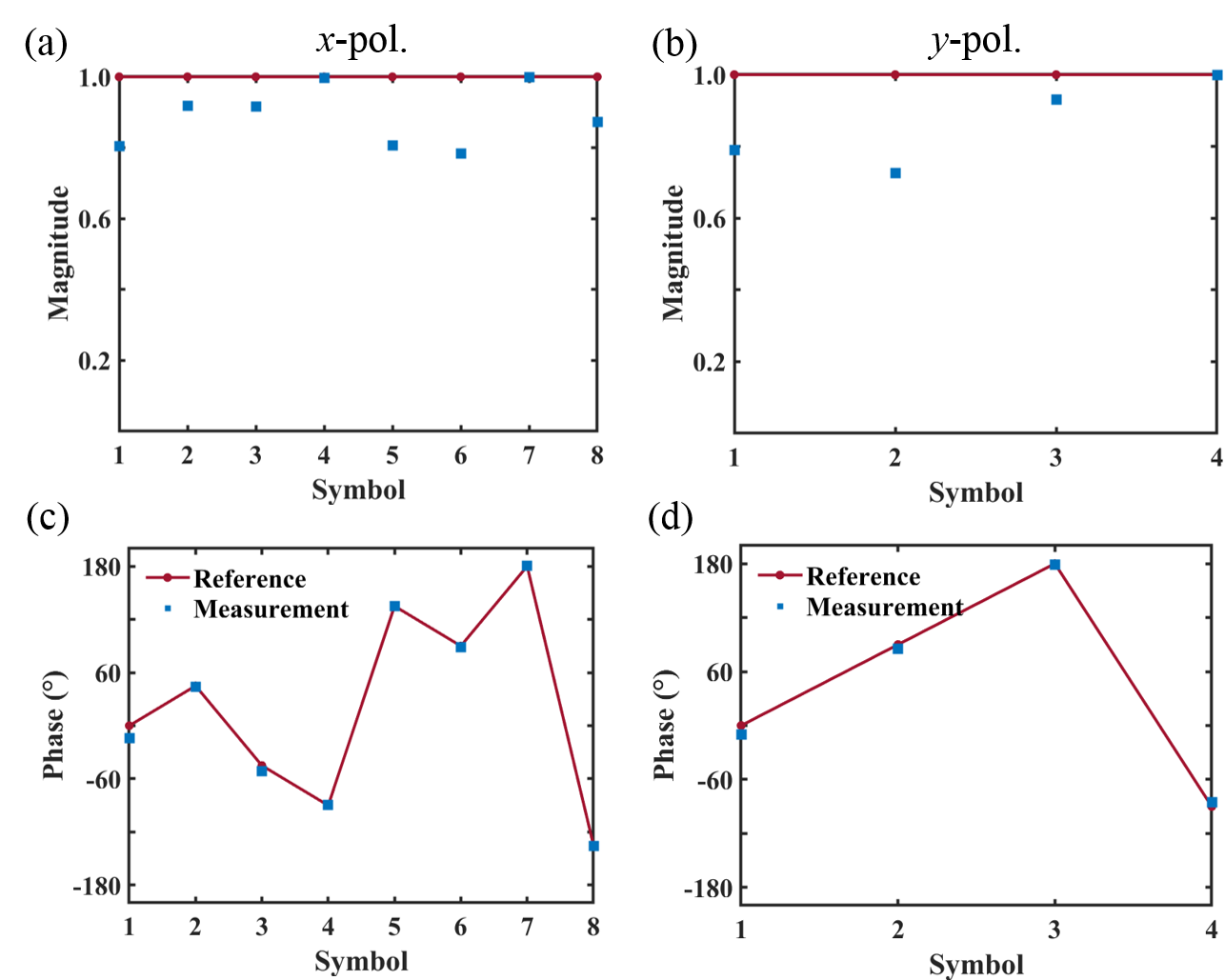


**Figure S10.** Amplitude and phase of the measured and reference signals in the user direction of $(\theta_{1}, \varphi_{1})=(-30^{\circ}, 180^{\circ})$. (a) Amplitude of the 8PSK symbols for the *x*-polarized channel. (b) Amplitude of the QPSK symbols for the *y*-polarized channel. (c) Phase of the 8PSK symbols for the *x*-polarized channel. (d) Phase of the QPSK symbols for the *y*-polarized channel.

**Note 12. Constellation diagrams in other directions for single-user mode**

To demonstrate the directional nature of the communication scheme, the constellation diagrams in other illegal directions are given in Figure S11. It can be obtained that the received signals are distorted.

**
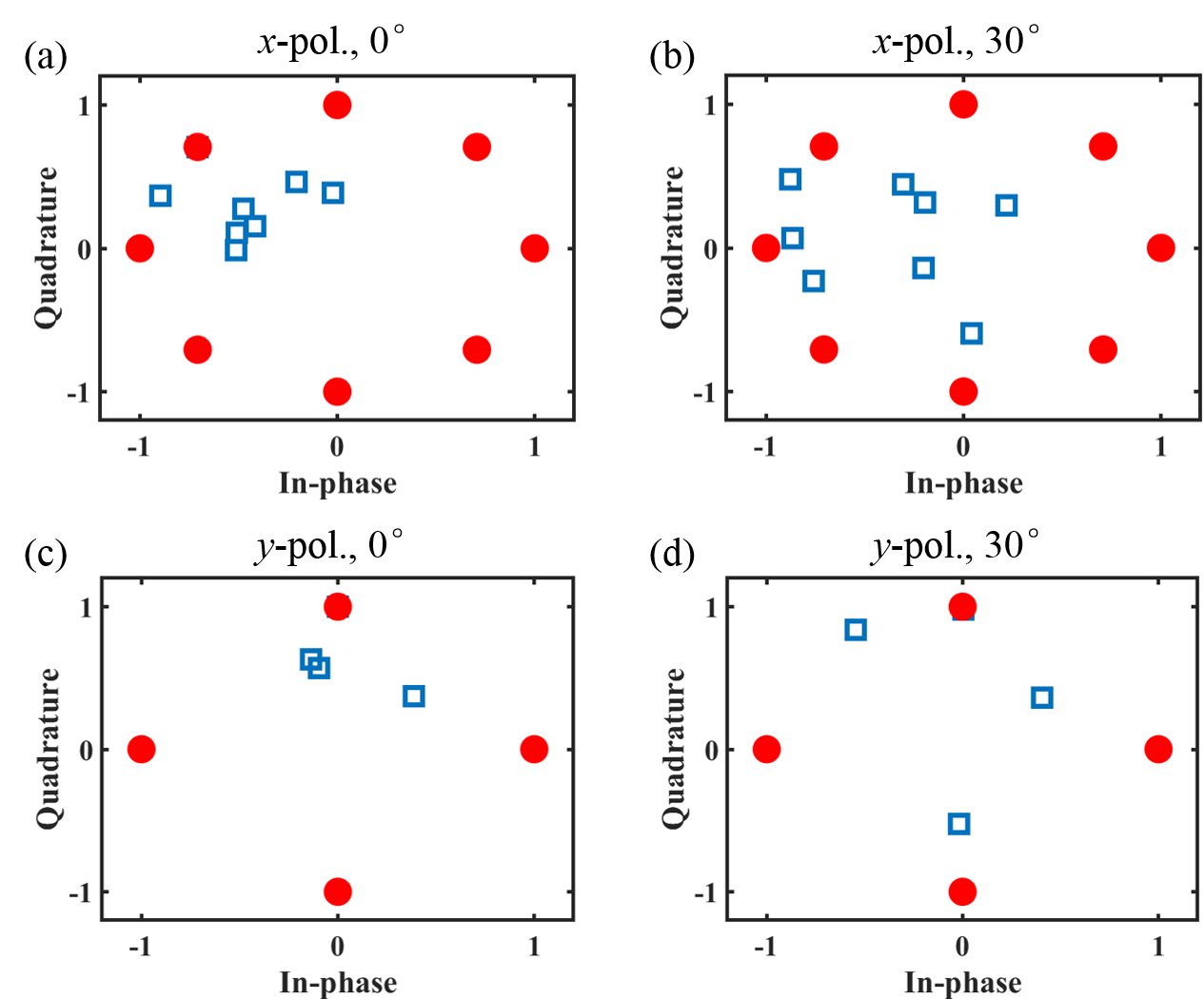
**

**Figure S11.** Constellation diagrams in other illegal directions for single-user mode. (a-b) Constellation diagrams in the directions of $(0^{\circ}, 0^{\circ})$ and $(30^{\circ}, 0^{\circ})$ for the *x*-polarized channel. (c-d) Constellation diagrams in the directions of $(0^{\circ}, 0^{\circ})$ and $(30^{\circ}, 0^{\circ})$ for the *y*-polarized channel.

**Note 13. Definition of EVM**

EVM can be used to measure signal quality in wireless systems. It is the vectorial difference between the actual signal and the reference signal at a given time, which provides a comprehensive measure of the amplitude and phase errors of the modulated signal. It is defined as follows

$\mathrm{EVM} (\mathrm{dB})=10{log}_{10} (P_{error}/P_{ref})=10{log}_{10} (\sqrt{\sum_{i=1}^{N} \left| \boldsymbol{s}_{ref}^{i}-\boldsymbol{s}_{mea}^{i} \right|^{2}/\sum_{i=1}^{N} \left| \boldsymbol{s}_{ref}^{i} \right|^{2}}$, (S7)

where $\boldsymbol{s}_{ref}$, $\boldsymbol{s}_{mea}$, and $N$ are the reference signal, the measured signal, and the number of symbols, respectively. From equation (S7), it can be seen that the smaller the EVM value, the closer the measured signal is to the reference.

**Note 14. Relationship of EVM values with** $\boldsymbol{\theta}$ **and** $\boldsymbol{\varphi}$

The relationship of EVM values with $\theta$ and $\varphi$ has been calculated, and the results are shown in Figures S12 and S13. It can be seen the EVM is sufficiently low only in the vicinity of the intended direction for both single- and dual-user modes.


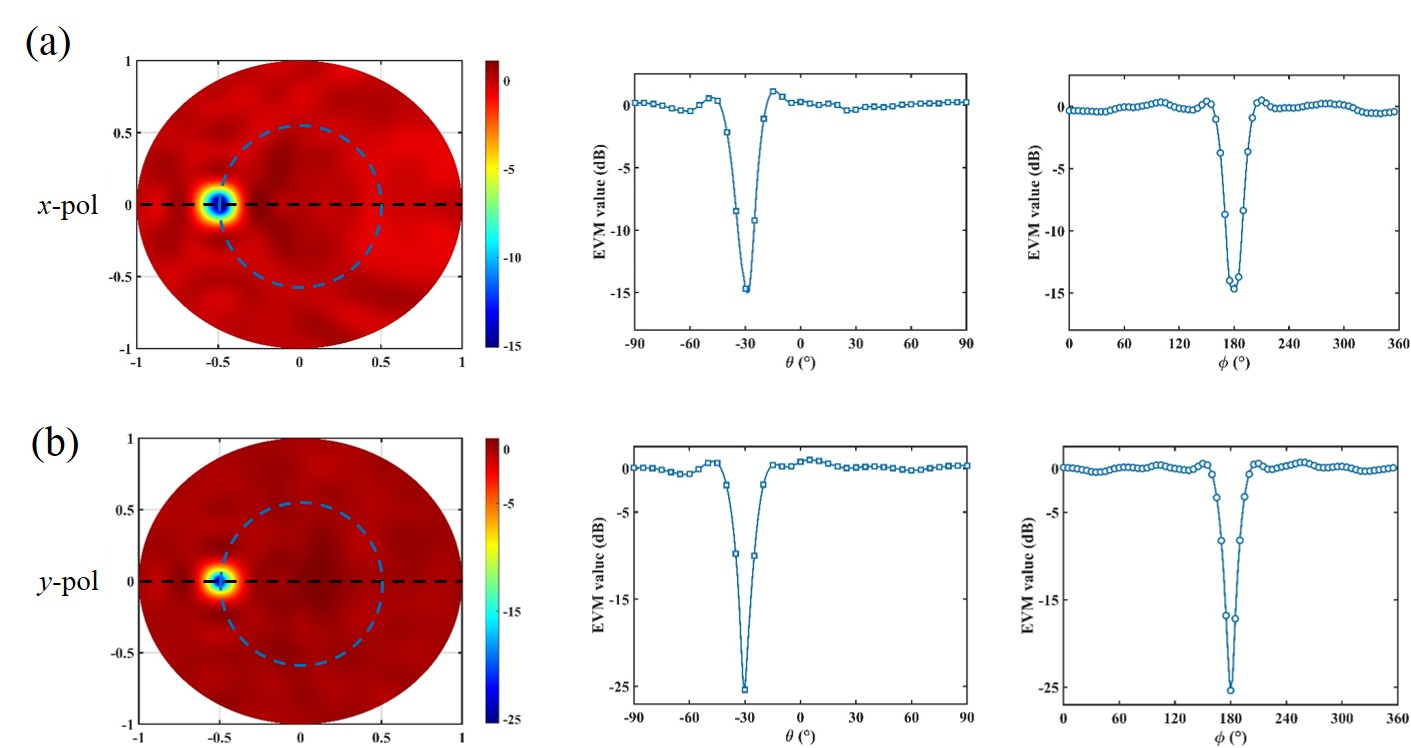


**Figure S12.** The relationship of EVM values in single-user mode. (a) *x*-polarized channel. (b) *y*-polarized channel.


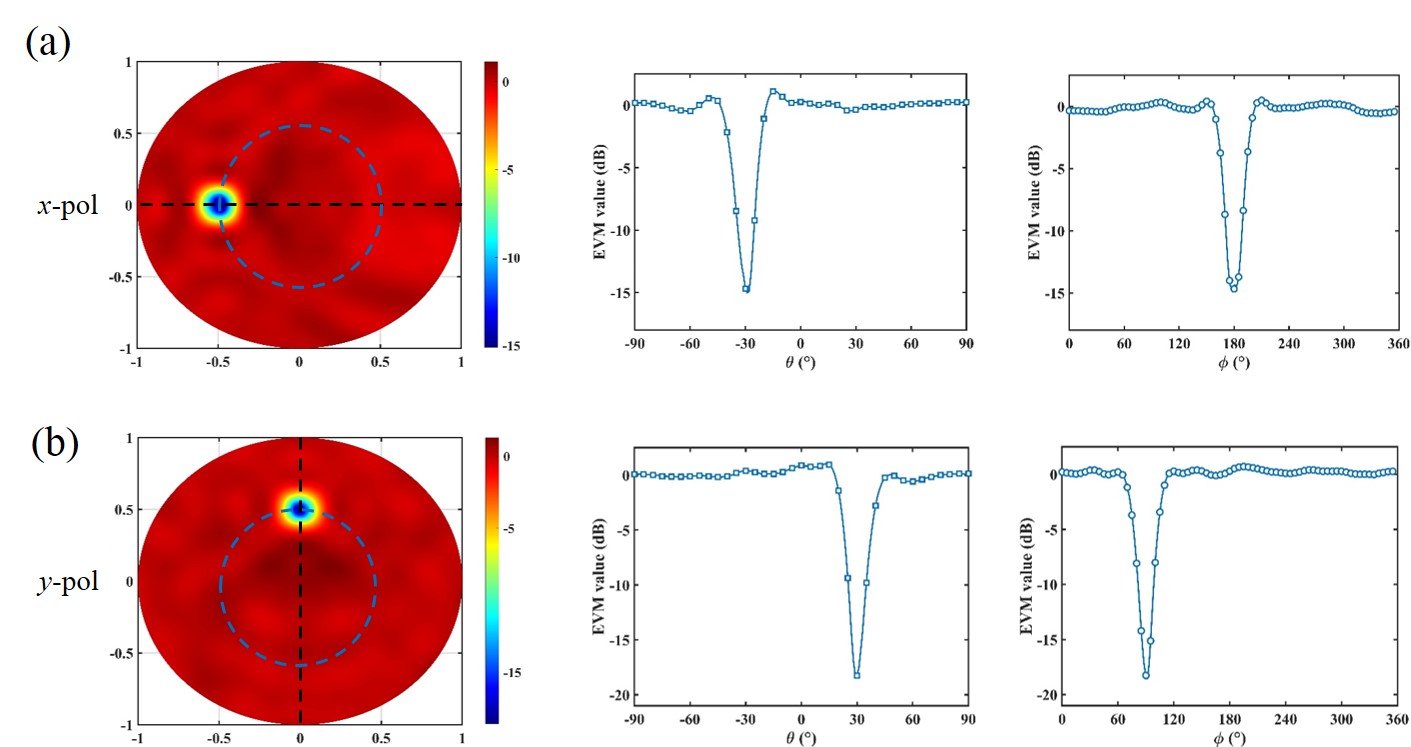


**Figure S13.** The relationship of EVM values in dual-user mode. (a) *x*-polarized channel. (b) *y*-polarized channel.

**Note 15. The measurement and reference signals for dual-user mode**

In dual-user mode, the amplitude and phase of the measurement and reference signals in the user directions of $(\theta_{1}, \varphi_{1})=(-30^{\circ}, 180^{\circ})$ and $(\theta_{2}, \varphi_{2})=(30^{\circ}, 90^{\circ})$ are plotted in Figure S14. It can be seen that the measurements are in good agreement with the reference.


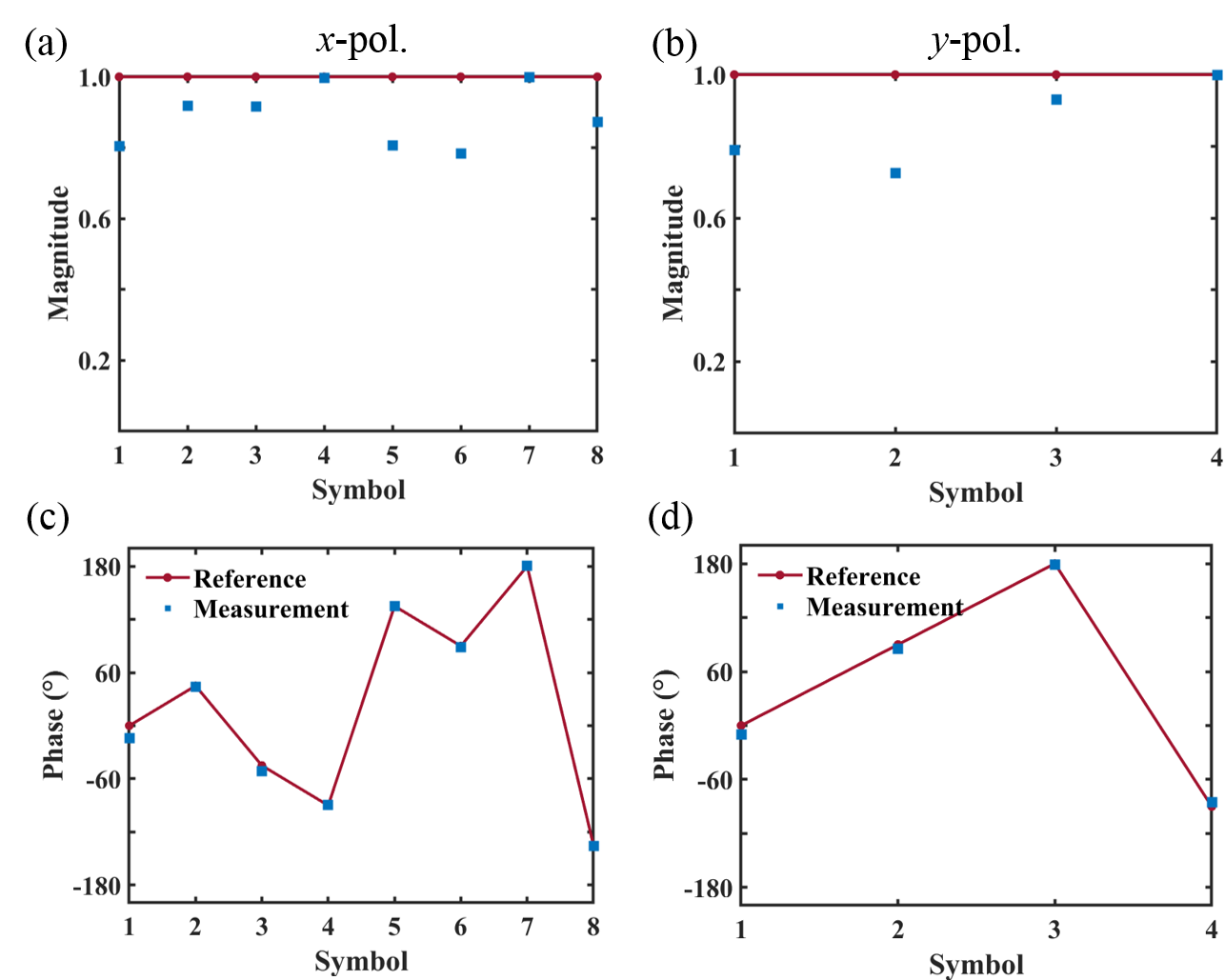


**Figure S14.** Amplitude and phase of the measured and reference signals in the user direction of $(\theta_{1}, \varphi_{1})=(-30^{\circ}, 180^{\circ})$ and and $(\theta_{2}, \varphi_{2})=(30^{\circ}, 90^{\circ})$. (a) Amplitude of the 8PSK symbols for the *x*-polarized channel. (b) Amplitude of the QPSK symbols for the *y*-polarized channel. (c) Phase of the 8PSK symbols for the *x*-polarized channel. (d) Phase of the QPSK symbols for the *y*-polarized channel.

**Note 16. Constellation diagrams in other directions for dual-user mode**

To demonstrate the directional nature of the communication scheme, the constellation diagrams in other illegal directions are given in Figure S15. It can be obtained that the received signals are totally distorted.

**
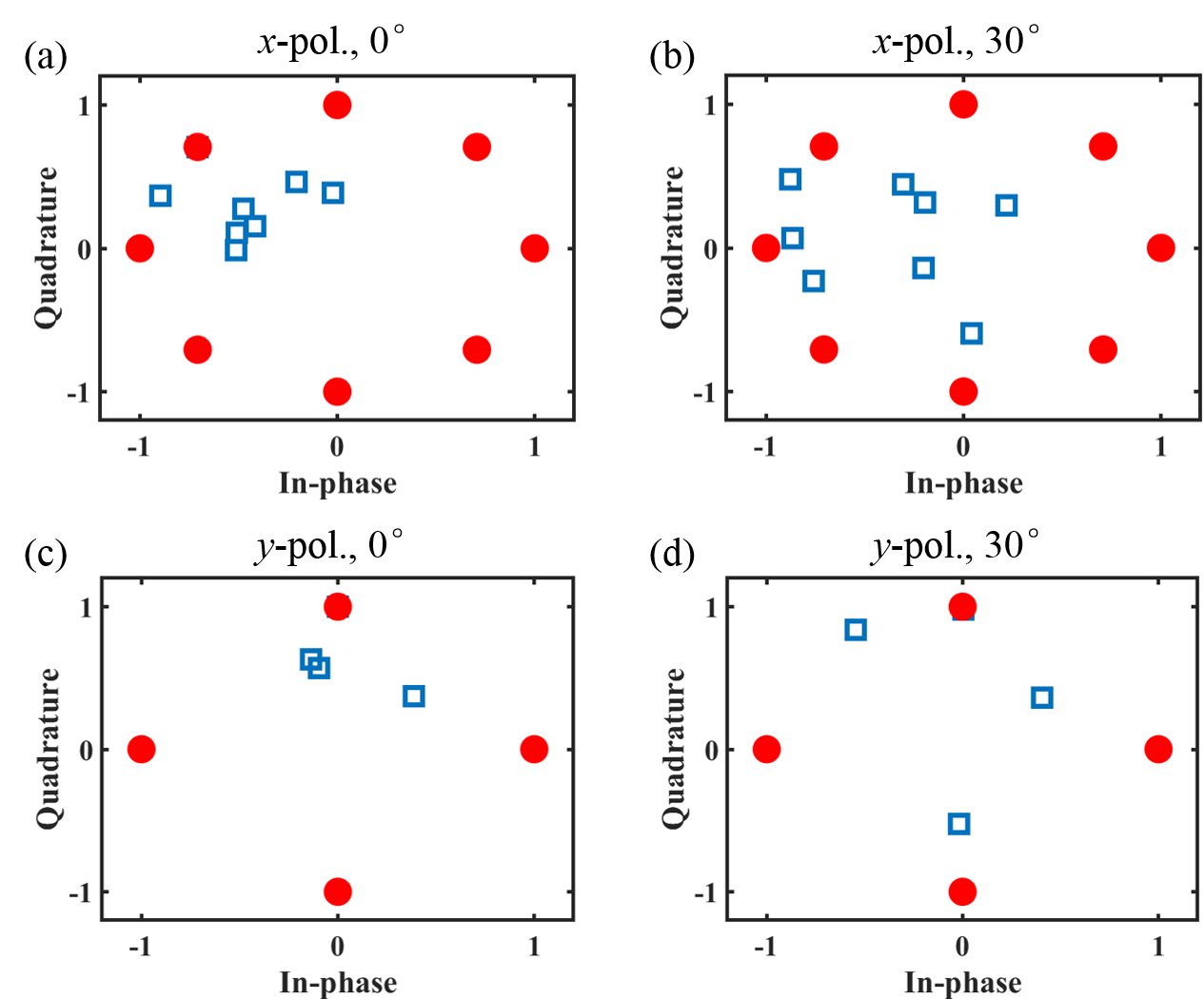
**

**Figure S15.** Constellation diagrams in other illegal directions for dual-user mode. (a-b) Constellation diagrams in the directions of $(0^{\circ}, 0^{\circ})$ and $(30^{\circ}, 0^{\circ})$ for the *x*-polarized channel. (c-d) Constellation diagrams in the directions of $(0^{\circ}, 90^{\circ})$ and $(30^{\circ}, 90^{\circ})$ for the *y*-polarized channel.
